# Supplementary figures and images for: Chronic replication stress-mediated genomic instability disrupts placenta development in mice
Source: PLoS Genet. 2026 Apr 13;22(4):e1012111. doi: 10.1371/journal.pgen.1012111 (PMC13099090; doi:10.1371/journal.pgen.1012111)

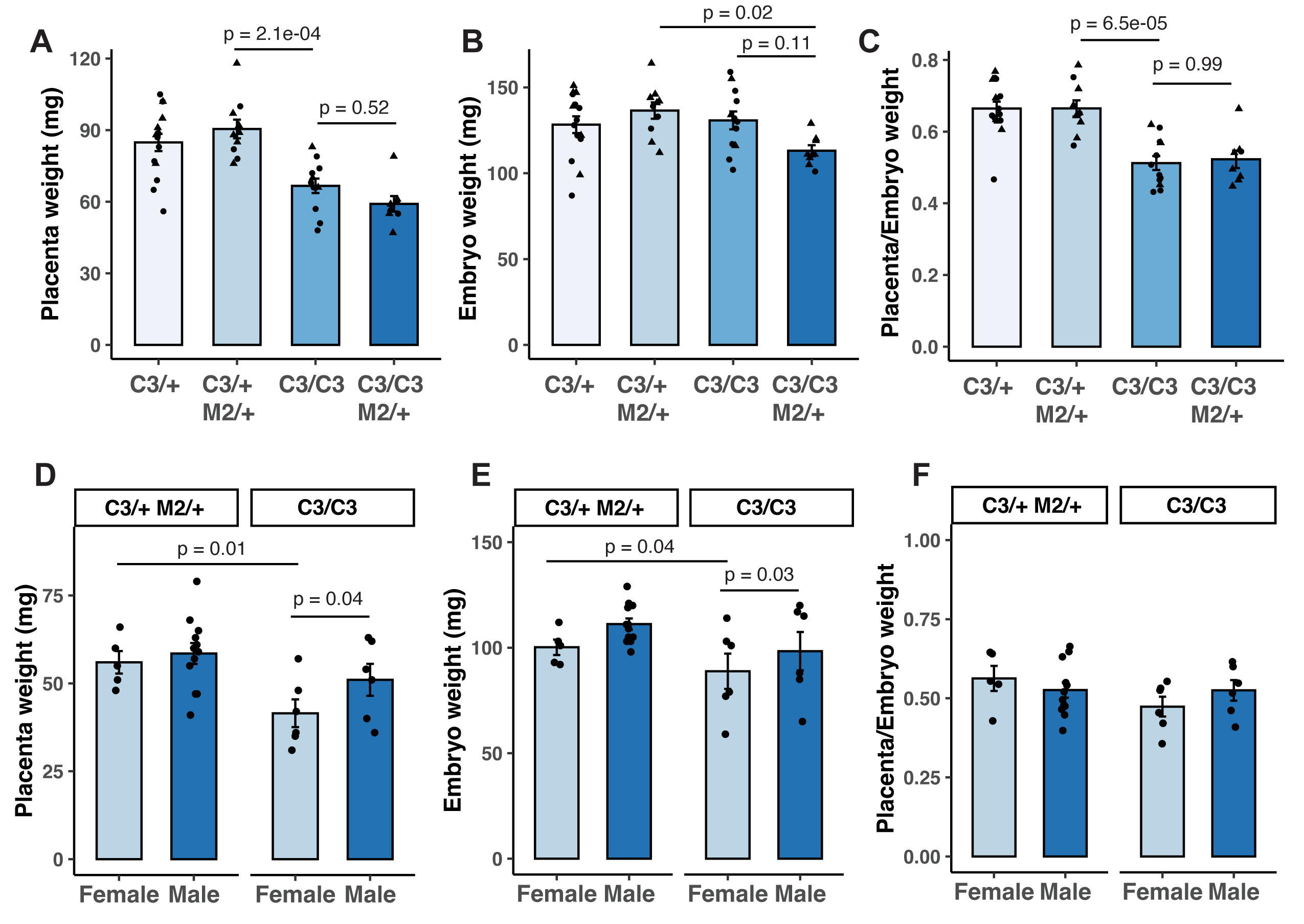

Supplement: S1 Fig — (A-C) Placental and embryonic weight as well as placental-to-embryonic weight ratio of each genotype from the reciprocal mating at E13.5. Females (circles), males (triangles). (D-F) Comparison of placental and embryonic weight as well as placental-to-embryonic weight ratio of male and female Mcm4C3/C3 Mcm2Gt/+ genotype from sex-skewing and reciprocal matings. Boxes indicate the maternal genotype. C3/ + : Mcm4C3/+; C3/C3: Mcm4C3/C3; C3/ + M2/ + : Mcm4C3/+ Mcm2Gt/+; C3/C3 M2/ + : Mcm4C3/C3 Mcm2Gt/+. p-values in (A-C) were calculated with one-way ANOVA followed by Tukey’s HSD test. p-values in (D-F) were calculated with two-way ANOVA. ns: not significant. *: p < 0.05; **: p < 0.01; ***: p < 0.001. Error bar: standard error of the mean. Each data point represents a single placenta or embryo. (TIF) [file pgen.1012111.s001.tif]

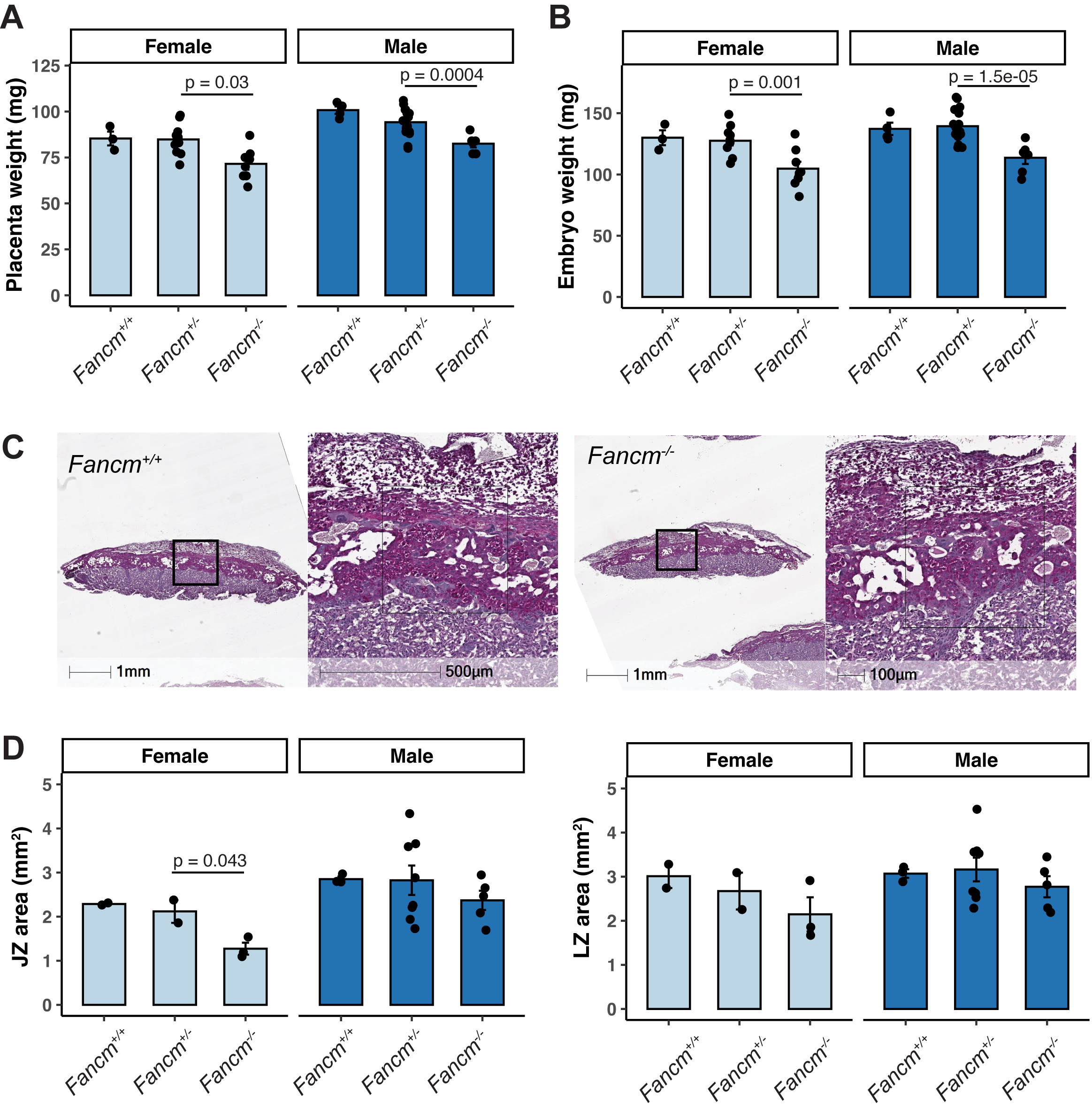

Supplement: S2 Fig — (A-B) Placental and embryonic weights of indicated genotypes at E13.5. (C) Periodic Acid Schiff staining of E13.5 placental sections from indicated genotypes. (D) Measurements of placental JZ and LZ areas. p-values were calculated with one-way ANOVA followed by Tukey’s HSD test. ns: not significant. *: p < 0.05; **: p < 0.01; ***: p < 0.001. Error bar: standard error of the mean. Each data point represents a single placenta or embryo. (TIF) [file pgen.1012111.s002.tif]

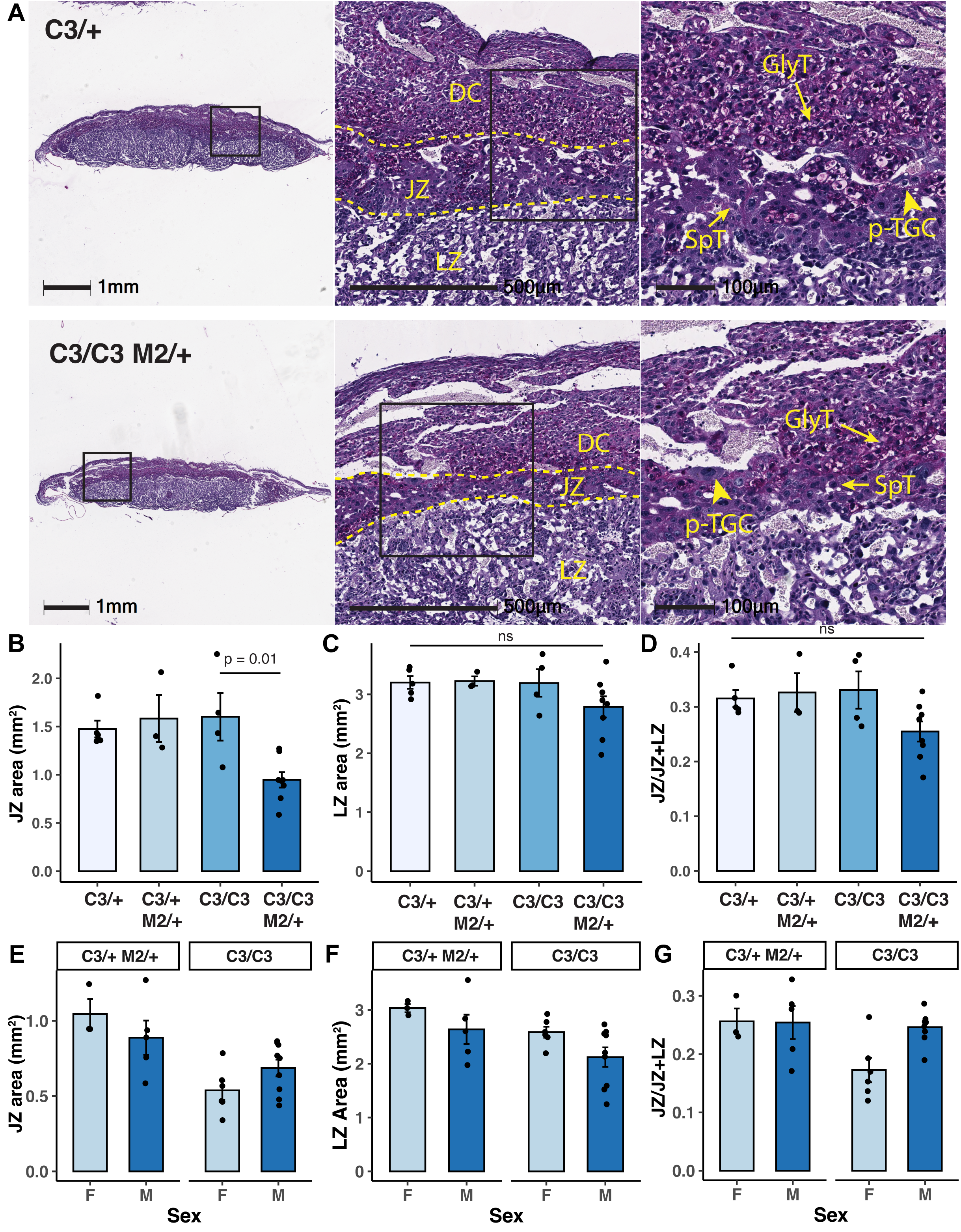

Supplement: S3 Fig — (A) Periodic Acid Schiff staining of placental sections from reciprocal matings at E13.5 for the indicated genotypes. (B-D) Measurements of placental JZ and LZ areas as well as the proportion of JZ from all indicated genotypes obtained from reciprocal matings at E13.5. (E-G) Comparison of JZ and LZ area as well as proportion of JZ in Mcm4C3/C3 Mcm2Gt/+ genotype from sex-skewing and reciprocal matings. Boxes above each graph indicate the maternal genotype. WT: wild type; C3/ + : Mcm4C3/+; C3/C3: Mcm4C3/C3; C3/ + M2/ + : Mcm4C3/+ Mcm2Gt/+; C3/C3 M2/ + : Mcm4C3/C3 Mcm2Gt/+. DC: decidua; JZ: junctional zone; LZ: labyrinth zone; p-TGC: parietal trophoblast giant cells; SpT: spongiotrophoblast; SynT: syncytiotrophoblast; GlyT: glycogen trophoblast; M: Male; F: Female. p-values were calculated with one-way ANOVA followed by Tukey’s HSD test. ns: not significant. *: p < 0.05; **: p < 0.01; ***: p < 0.001. Error bar: standard error of the mean. Each data point in (B-G) represents the average measurement taken from at least three sections of the same placenta. (TIF) [file pgen.1012111.s003.tif]

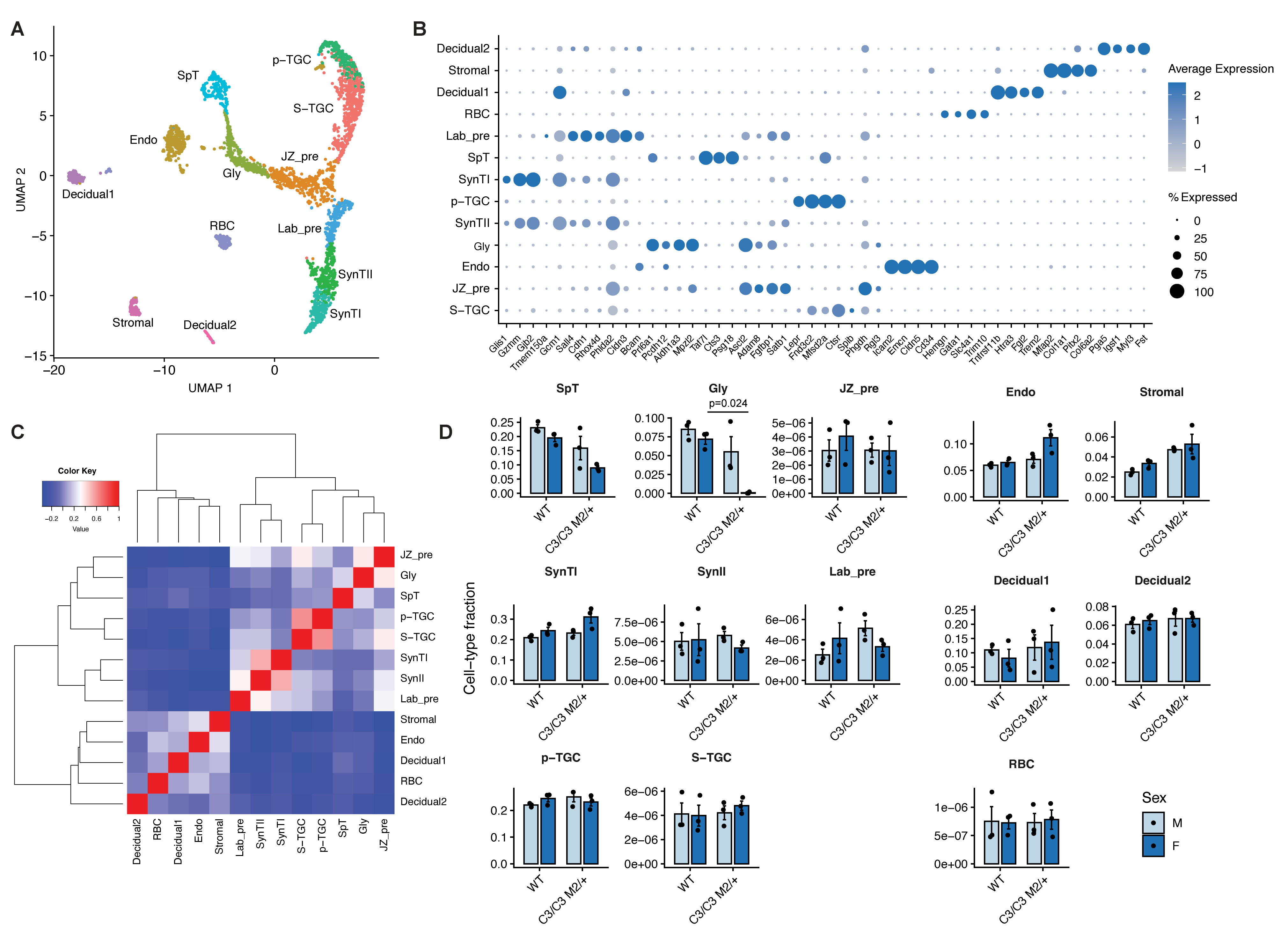

Supplement: S4 Fig — (A) UMAP of E13.5 placental single-cell RNA-Seq. (B) Gene expression dot plot of marker genes for different cell types from reference placental scRNA-Seq. (C) Pairwise correlation of cell types based on the single cell transcriptome. (D) Cell type fractions identified by BayesPrism in control and semi-lethal genotype placentae. WT: wild type; C3/C3 M2/ + : Mcm4C3/C3 Mcm2Gt/+. SpT: spongiotrophoblast; Gly: glycogen trophoblast; JZ_pre: junctional zone precursors; SynTI: syncytiotrophoblast I; SynTII: syncytiotrophoblast II; Lab_pre: labyrinth precursors; p-TGC: parietal trophoblast giant cells; S-TGC: sinusoidal trophoblast giant cells; Endo: endothelial cells; RBC: red blood cells. Each data point in (D) represents the cell type fraction calculated from one placental sample bulk RNA-Seq. Error bars in (D)represent standard error of the means, and each dot represents a single placental sample. *: q < 0.05 (Benjamini–Hochberg FDR adjusted q-value). (TIF) [file pgen.1012111.s004.tif]

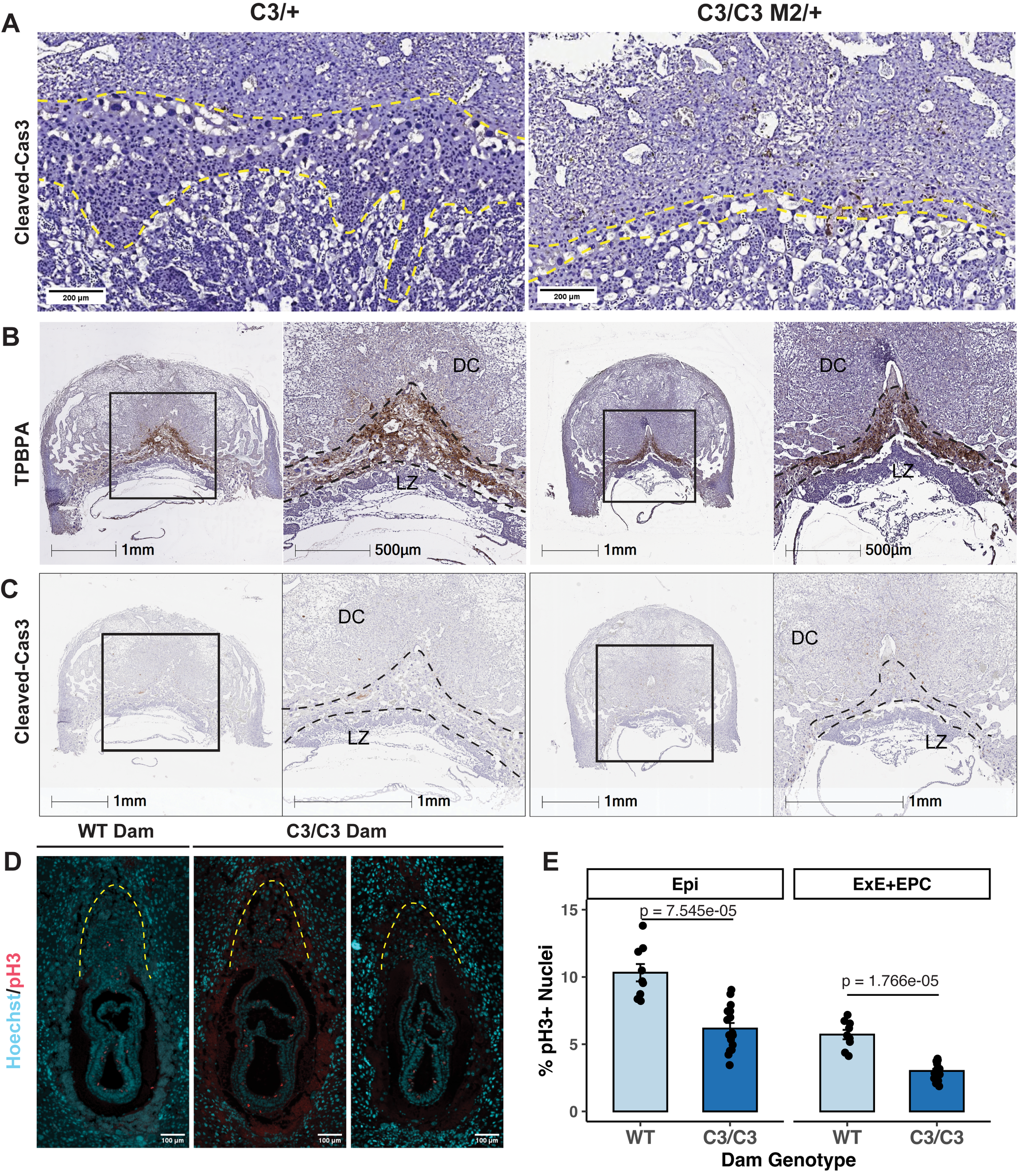

Supplement: S5 Fig — (A) Representative images of Cleaved-caspase 3 immunohistochemistry in E11.5 placental sections for the indicated genotypes. (B-C) Representative images of TPBPA and Cleaved-caspase 3 immunohistochemistry in E9.5 placental sections for the indicated genotypes. (D) Representative images of phospho-H3 immunofluorescence in E7.5 embryos sections. (E) Quantification of phospho-H3 positive cells in the epiblast and extraembryonic ectoderm. WT: wild type; C3/ + : Mcm4C3/+; C3/C3: Mcm4C3/C3; C3/C3 M2/ + : Mcm4C3/C3 Mcm2Gt/+. Epi: epiblast; ExE: extraembryonic ectoderm; EPC: ectoplacental cone; DC: decidua; LZ: labyrinth zone. Each data point in (E) represents the average percent positive cells from at least three consecutive sections of a single embryo. (TIF) [file pgen.1012111.s005.tif]

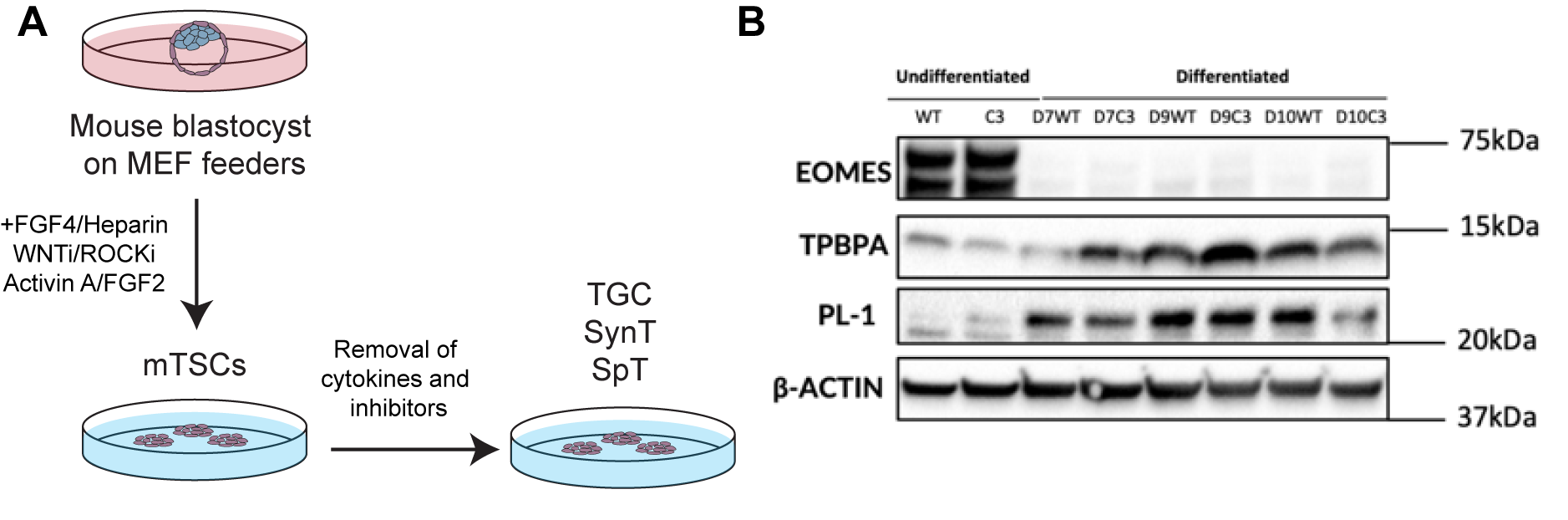

Supplement: S6 Fig — (A) Schematic representation of TSC derivation from E3.5 blastocysts under defined culture conditions. (B) Western blot analysis of trophoblast markers before and after TSC differentiation. Genotypes are indicated: C3, Mcm4Chaos3/Chaos3; D, day; PL-1 and TPBPA are markers of TGCs and spongiotrophoblast, respectively. β-actin is a loading control. (TIF) [file pgen.1012111.s006.tif]

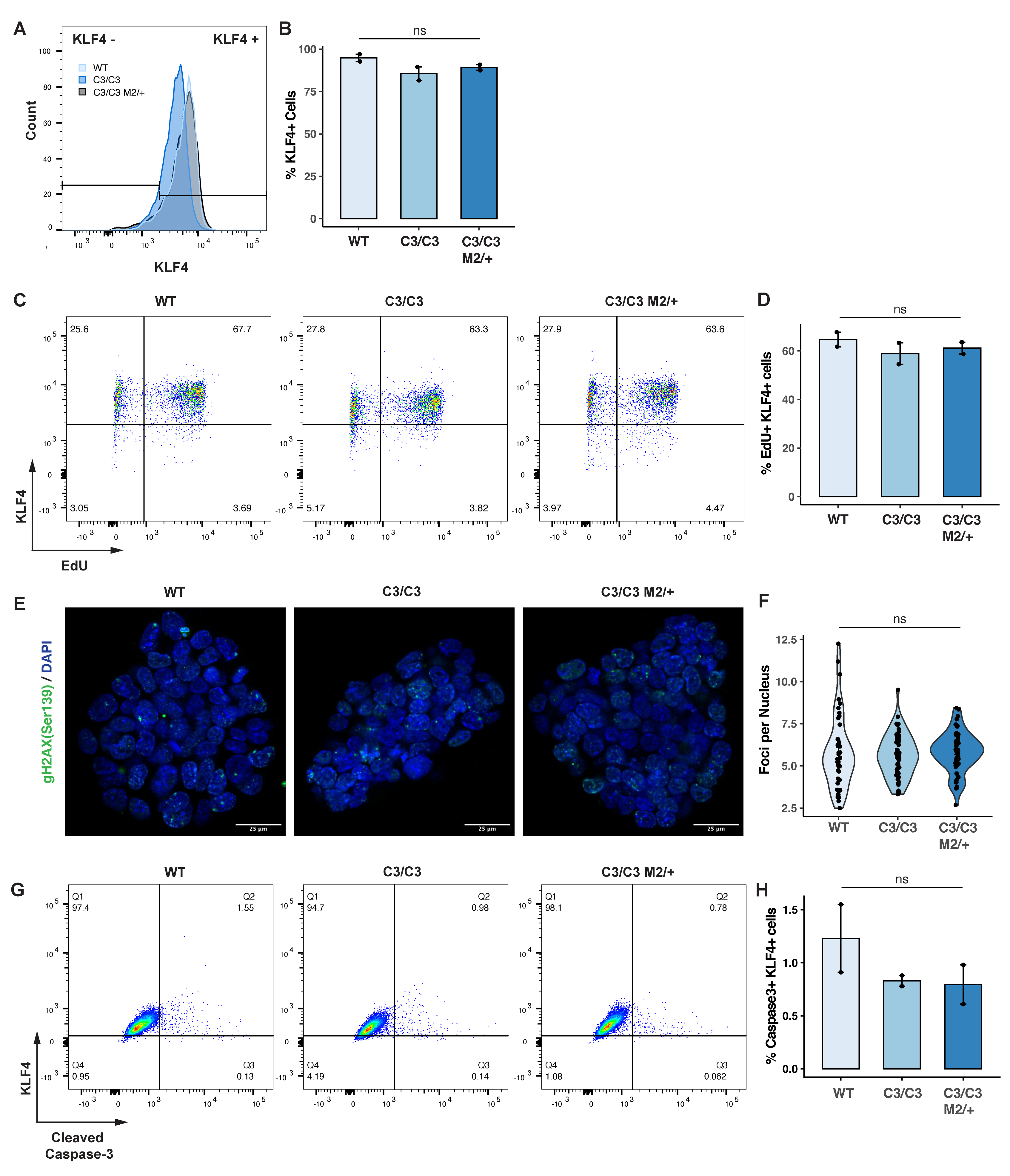

Supplement: S7 Fig — (A) Flow cytometry analysis of naive pluripotency marker KLF4 in WT, C3/C3, and C3/C3 M2/ + ESCs. (B) Quantification of KLF4 positive cells in ESCs. (C) Flow cytometry analysis of EdU pulse labeled and KLF4 stained ESCs in indicated genotypes. (D)Quantification of the EdU and KLF4 double positive cells from flow cytometry. (E)Immunofluorescent analysis ofγH2AX in ESCs of the indicated genotypes. (F) Quantification of γH2Ax foci per nuclei in each indicated genotype. Each data point in (B), (D), (H) represents two technical replicates; dots in (F) represents foci per nuclei analyzed from individual images from two technical replicates. Error bar: standard error of the means. ns: not significant; p-values were calculated using one-way Anova. (TIF) [file pgen.1012111.s007.tif]

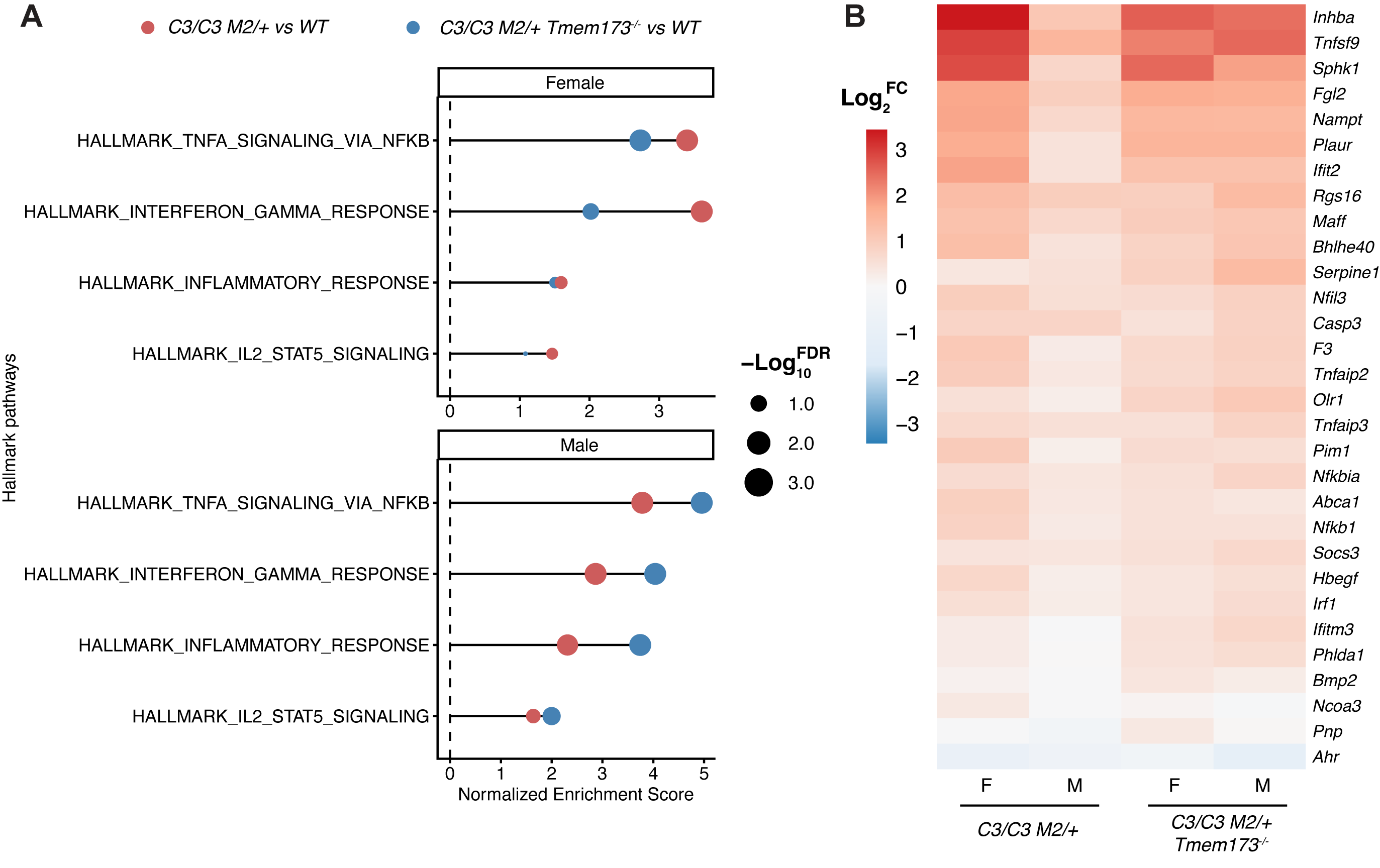

Supplement: S8 Fig — (A) GSEA analysis of bulk RNA-Seq data from E13.5 placentas collected from Mcm4C3/C3 Mcm2Gt/+ and Mcm4C3/C3 Mcm2Gt/+ Tmem173-/- genotypes. Normalized enrichment scores of Hallmark inflammatory pathways in Mcm4C3/C3 Mcm2Gt/+ or Mcm4C3/C3 Mcm2Gt/+ Tmem173-/- compared to the wild type were plotted for both sexes. (B) Expression of genes involved in inflammatory pathways identified using leading edge analysis in male and female semi-lethal and STING-deficient semi-lethal genotype placentas compared to wild type, respectively. (TIF) [file pgen.1012111.s008.tif]
