# Supplementary material for: Chronic replication stress-mediated genomic instability disrupts placenta development in mice
Source: PLoS Genet. 2026 Apr 13;22(4):e1012111. doi: 10.1371/journal.pgen.1012111 (PMC13099090; doi:10.1371/journal.pgen.1012111)
Supplement: Attachment — Submitted filename: 158064_1_rebuttal_3530818_t3grpc.docx [file pgen.1012111.s015.docx]

**Manuscript number:** RC-2025-03034R

**Corresponding author(s):** John, Schimenti

*[The “revision plan” should delineate the revisions that authors intend to carry out in response to the points raised by the referees. It also provides the authors with the opportunity to explain their view of the paper and of the referee reports.*

*The document is important for the editors of affiliate journals when they make a first decision on the transferred manuscript. It will also be useful to readers of the reprint and help them to obtain a balanced view of the paper.*

*If you wish to submit a full revision, please use our "*[*Full Revision*](https://reviewcommons.org/templates/full-revision.docx)*" template.* ***It is important to use the appropriate template to clearly inform the editors of your intentions.****]*

## 1. General Statements [optional]

*This section is optional. Insert here any general statements you wish to make about the goal of the study or about the reviews.*

We thank the reviewers for providing thoughtful and constructive feedback, which will help us improve the clarity and rigor of the paper. On balance, the reviews were positive. Reviewer 1 mentioned that “This is a strong manuscript with few problems and all important findings well justified, indeed this is a nicely polished…..high-quality manuscript,” and that “this paper makes a major breakthrough, showing that cell autonomous defects in hTSCs are very likely at the heart of the pathology observed in GIN-prone murine mutants.” Reviewer 3 stated that “The study is well designed, and the manuscript is very well written. The conclusions are supported by the evidence presented.” Reviewer 2 was less enthusiastic, with main concerns being that “The paper is mostly descriptive and often quite confusing leaving one not much closer to understanding the mechanistic basis for the interesting sex-biased semi-lethal phenotype.” and felt that figure titles/section headers overstated the results, and finally recommended to improve some technical aspects and tempering conclusions. The proposed edits we think address most issues raised by the reviewers either with re-writing or adding data as described below.

## 2. Description of the planned revisions

*Insert here a point-by-point reply that explains what revisions, additional experimentations and analyses are planned to address the points raised by the referees.*

**In response to reviewer #1 comments:**

*Major comments:*

- I am confused as to the basis of the sex-skewing phenomenon? Is the problem that lack of maternally loaded WT Mcm4 worsens the phenotype, or is the issue that Mcm4C3/C3 dams are less able to retain pregnancies, perhaps being a more inflammatory environment? Also, while there quite consistent evidence for reduced viability of Mcm4C3/C3McmGt/+ progeny, especially for female progeny, how confident can we be that the genotype of the dam vs. sire is important? Notably on a Ddx58 background, the progeny of the Mcm4C3/C3 sire included seven live male Mcm4C3/C3McmGt/+ but no female.

Regarding the first point (sex skewing only when female is C3/C3), we also suspected either: 1) the maternal uterine environment, or 2) reduced oocyte quality. Although not reported in this manuscript, we tested #1 by performing embryo transfer experiments. Transferring 2-cell stage embryos from sex-skewing mating to WT females did not rescue the sex-bias. We then examined oocytes from C3/C3 females. We found evidence for compromised mitochondria and transcriptome disruption. However, we are not sure why this happens (poor follicle support? Oocyte intrinsic phenomenon?). We are reserving these results and additional experiments for another paper, especially since this one mainly deals with GIN and placenta development. If the reviewers feel strongly that the embryo transfer data is crucial, we can include it.

Regarding how confident we are that the genotype of the dam vs. sire is important, this stems from our previous paper by McNairn et al 2019 (the percentage of female C3/C3 M2/+ from sex-skewing mating is 20% compared to 60% from the reciprocal mating), which was quite dramatic. Consistent with this, MCM levels were significantly reduced in the placentae only when the dam was C3/C3 and the sire C3/+ M2/+, but not in the reciprocal cross. The reviewer makes a good observation about the Ddx58 cross; we can only hypothesize that the mutation somehow sensitizes females in this scenario and will make mention of it in the revision. We also realize that we neglected to write in Methods that the Ddx58 allele was coisogenic in the C3H background.

- I'm not sure what Supplementary Figure 6 is showing (faster differentiation of C3 but less TGC?). Regardless, it's hard to draw too much conclusion from one not-very-pretty Western blot. This figure requires both additional replicates and a better explanation of how it fits with the other conclusions of the paper..

We hypothesized that the JZ defect observed in the semi-lethal genotype placentas could arise either from impaired maintenance of the progenitor pool or from reduced capacity of mutant trophoblast progenitors to differentiate into the JZ lineage. The blot in Supplementary Figure 6 was intended as a qualitative demonstration that mutant trophoblast stem cells can differentiate into JZ lineages. We recognize that the figure is not definitive and will revise the text to clarify its purpose. A replicate(s) of the Western will be performed as suggested.

- Supplementary Figure 7F-G is puzzling. Half of the mESCs have gamma-H2AX at all times, including most in S or G2 phase? In Figure S7E, do the quadrants correspond to being negative or positive for gamma-H2AX? At very least, IF images showing clear gamma-H2AX foci would be much more convincing.

The gates for γH2AX FACS analysis were established using negative controls lacking primary antibody. As reported previously, embryonic stem cells display high basal levels of γH2AX staining (Chuykin et al., Cell Cycle 2008; Turinetto et al., Stem Cells 2012; Ahuja et al., Nat Comm 2016), which likely explains the broad signal observed across cell cycle phases. Regardless, we will provide immunofluorescence staining of γH2Ax and foci count in our revision.

- The methods section is well detailed, but it would be ideal to clarify how many replicates each Western Blot or flow cytometry experiment is representative of.

Thanks for the suggestion. We will update this for Fig4 and Fig5.

*Minor comments:*

- Is it possible that cGAS-STING and RIG pathways act redundantly to cause inflammation and lethality, or that other innate immune components are involved? I don't expect the authors to make compound mutants to test this but at least this possibility should be discussed textually.

We appreciate the reviewer’s point, and had the same suspicion. Supporting this, we will add new RNA-seq analysis of Tmem173 KO placentas revealed elevated inflammatory gene expression compared to C3/C3 M2/+ controls, consistent with potential redundancy or feedback regulation. We will update in supplementary figures to reflect this.

2. **In response to reviewer #2 comments:**

*Major comments:*

A major concern throughout the paper is that conclusions are often overstating their data. The title of figure 2 is "placentae with replication stress have smaller junctional and labyrinth zones". However, there is no measure of replication stress in this figure, just a histological evaluation of the placentae from the different mutants. The title of figure 3 is "Impact of GIN on LZ is less than JZ," but there is no measure of GIN, but instead measurement of number of cells in cell cycle and some bulk RNA-seq analysis. Title of figure 4 is "TSCs with increased genomic instability exhibit abnormal phenotypes." Again there is no measure of GIN, but instead staining of derived TSCs for proliferation, cell death, and a TSC marker. Title of figure 5 is "DNA damage responses and G2/M checkpoint activation drive premature TSC differentiation." However, there does not appear to be a difference in gH2AX between the two mutant genotypes. Checkpoint proteins might be up, but need quantification and reproduction. > 4C is the only marker of differentiation. Importantly, all the analyses here are associations, not connections, so cannot use the word "drive". Similar issues can be raised with a number of the supplementary figures.

The *Chaos3* (chromosome aberrations occurring spontaneously 3) model is a well-established system of intrinsic chronic replication stress and GIN. It is characterized by ~20 fold elevation of blood micronuclei (Shima et al., Nature 2007), a hallmark of GIN (Soxena et al., Mol Cell 2022); a destabilized MCM2-7 helicase prone to replication fork collapse (Bai et al., PLoS Genet 2016); and increased mitotic chromosome abnormalities and decreased dormant origins (Kawabata et al., Mol Cell 2011; Chuang et al., Nucleic Acid Res 2012) that are known to cause GIN and replication stress (Ibarra et al., PNAS 2008 ). Also, in our previous work (McNairn et al Nature 2019), we showed that placentae from C3/C3 dams exhibit significantly elevated γH2Ax as well as reduced MCM2 and MCM4 protein levels. In our current study, we also observe elevated γH2Ax in mutant TSCs (C3/C3 and C3/C3 M2/+), consistent with genomic instability. Nevertheless, we acknowledge that in TSCs, we did not formally demonstrate replications stress(RS), so where appropriate, we will advise figure titles, for example to say that “cells/placentae with a GIN or RS genotype.”

We acknowledge the reviewers concern regarding western blots. We will provide quantification and statistics in our revision.

1)  A deeper analysis of the cell lines is likely to be the most fruitful path to reveal interesting mechanisms. It is very surprising that there is no phenotype in ESCs. Authors should check for increased apoptosis. Maybe the phenotypic cells are lost. Or do ESCs use different MCMs/mechanisms of DNA replication or are they better able to handle replication stress and GIN? How many passages were the TSCs and ESCs cultured for? Does GIN (i.e. aneuploidy, CNVs) develop in TSCs and ESCs with passaging? How do the MCM mutations impact the molecular identity of the ESC and TSC cells including their heterogeneity in the population.

We assessed apoptosis using cleaved caspase 3 flow cytometry in mutant ESCs and observed no difference compared to controls (we will add this data as Supplementary Fig. 7).

We believe there are intrinsic differences in TSCs and ESCs in their ability to respond to and counteract replication stress and DNA damage. ESCs are known to license more replication origins than somatic cells at a higher rate, which protects them from short G1-induced replication stress (Ahuja et al., Nat Comm 2016; Ge et al., Stem Cell Rep 2015; Matson et al., eLife 2017). Human placental cells physiologically exhibit high levels of mutation rate and chromosomal instability *in vivo* (Coorens et al., Nature 2021)*.* Supporting this, Wang, D., et al (Nat Comm 2025) reported that several cell cycle and DDR regulators are differentially expressed in human TSCs vs human pluripotent stem cells. Whether such transcriptional differences directly contribute to functional outcomes remains to be determined.

All experiments in this study were conducted using early-passage ESCs and TSCs (i.e. <10 passage) that we ourselves derived. Although we agree that examining mutation rates with extended passaging is of interest, and this is an ongoing area of research with clinical relevance, this is beyond the scope of the current work.

Finally, we showed that close to 90% mutant ESCs are KLF4+ (a naive pluripotency marker) whereas EOMES+ cells were significantly reduced in TSCs carrying the GIN genotype (Fig. 4E–F and Supplementary Fig. 7), highlighting lineage-specific differences.

*Minor Comments:*
1) There is a lack of quantification and repeats for all Westerns. At minimum there should be three repeats for each experiment, quantification including normalization to a reference protein, and stats confirming any proposed differences between conditions.

We will update our revision with quantification and statistics for western blots.

2) I would recommend moving the results in supp table 1 to figure 1. While negative, they are the newer results. The results shown in current figure 1 are essentially a reproduction of their previous work.

The placental observations presented in Fig.1 are new. In particular, the placental and embryonic weight measurements graphed in Fig1B and C have not been published by our group. Fig1A reproduces our previous observation on embryo viability in GIN mutants (McNairn et al., Nature 2019), while the schematic was provided for better flow and readability given the complex mating schemes. We are agnostic on the Suppl Table 1. It could be changed to a new Table 1 in the main section depending on the journal.

3. **In response to reviewer #3 comments:**

Major Comments

• While the inclusion of bulk RNAseq data of whole placental tissue is appreciated, the interpretation of the results is somewhat problematic, as it is acknowledged that the cell type composition of the placentas is drastically different between groups. Making conclusions based upon GSEA analysis of two different groups with drastically different cell type composition is somewhat misleading, as based on the results, it is a direct reflection of the cell types present. It would be more helpful to perform cell type deconvolution of the RNAseq data to estimate the proportion of each cell type within the bulk samples and compare that to what is seen histologically and not dive too deeply into the pathways since the results could just be a reflection of the cell types e.g. angiogenesis pathways from more endothelial cells. Additionally, the RNAseq data can be leveraged to look at expression of inflammatory genes by sex, which may show interesting patterns based on the other results.

We agree that the representation of cell types in the placenta is problematic especially for underrepresented genes. We propose to use the BayesPrism tool (Chu et al., Nat Cancer 2022) to deconvolute bulk RNA-seq for better representation of transcriptional changes in the placenta.

• Section: GIN impairs trophoblast stem cell establishment and maintenance. To support the assertion in the first paragraph, beyond measuring apoptosis, it would be helpful at this stage to look at RNA expression levels indicative of the activation of DNA damage checkpoint genes

We have performed RNA-seq on mutant ESC and TSCs and are in the process of data analysis. We will update these results in the revision.

• Please include additional methodological details in the methods section on the statistical analysis done for differential expression analysis. Specifically, what type of normalization was used, if lowly expressed genes were filtered out and at what cutoff, what statistical model was used (did you include covariates?), what comparisons were made? Did you stratify by sex? What cutoff was used for statistical significance? Did you perform multiple testing correction?

We will update RNA-Seq data analysis methods in our full revision.

## 3. Description of the revisions that have already been incorporated in the transferred manuscript

*Please insert a point-by-point reply describing the revisions that were already carried out and included in the transferred manuscript. If no revisions have been carried out yet, please leave this section empty.*

*Reviewer #1 comments:*

- Supplementary Table 1. would be enhanced greatly showing comparable tables for Mcm4C3/C3 x Mcm4C3/+McmGt/+ in mice without the Tmem173 or Ddx58 mutations. It is fine to recycle data from McNairn 2019 here, as long as the source is indicated, but a comparison is needed.

Thanks for pointing this out. We have updated this suggestion in Supp table 1.

- In Figure S3E-F, is the box above each graph supposed to show the genotype of the dam?

Yes. Thanks for pointing this out. We have added a description in the figure legend to make it clear.

- "Indeed, the placenta and embryo weights of E13.5 Mcm4C3/C3 Mcm2Gt/+ Mcm3Gt/+ animals were significantly improved vs. Mcm4C3/C3 Mcm2Gt/+ animals, rendering them similar to Mcm4C3/C3 littermates (Fig. 6A-C). The JZ (but not LZ) area in Mcm4C3/C3 Mcm2Gt/+ Mcm3Gt/+ placentae also increased to the level of Mcm4C3/C3 littermates (Fig. 6D-H)."
There are two problems here. First, the figure calls are wrong. Second, the description of the data is not quite right, it looks like the C3/C3 and C3/C3 M2/+ M3/+ LZs are a similar size to each and are statistically indistinguishable.

Thanks for catching this. We have updated these in the main text.

*Reviewer #2 comments:*

*Minor comment*

3) Need to review citations to figures. For example, no citations are made to figure 4a and 4c.

Thanks for catching this. We have updated the text.

*Reviewer #3 comments:*

• Define the first use of >4C DNA content to help readers understand this potentially unfamiliar term.

We have edited this part to indicate cells with more than 4C DNA content for better clarity.

• iDEP tool - please include citation to manuscript instead of link

We have updated this citation.

• Check citations. Some citations to BioRxiv that are now published e.g. 13.

We have updated this citation.

## 4. Description of analyses that authors prefer not to carry out

*Please include a point-by-point response explaining why some of the requested data or additional analyses might not be necessary or cannot be provided within the scope of a revision. This can be due to time or resource limitations or in case of disagreement about the necessity of such additional data given the scope of the study. Please leave empty if not applicable.*

Reviewer 2

2) Along similar lines, most of the in vivo phenotypic analyses are performed at E13.5, long after defects are likely beginning to express themselves especially given that they see phenotypes in the TSCs, which represent the polar TE of a E4.5. To understand the primary defects of the in vivo phenotype, they should be looking much earlier. Supplemental figure 5 is a start but represents a rather superficial analysis.

The peri-implantation period, namely E4.5, represents a “black box” of embryonic development given that this is a critical stage for implantation. Aside from being an extremely difficult stage to analyze technically, we don’t think it is essential to the conclusions (or doable in a timely manner), especially given the use of TSCs. If we complete EdU studies on E6.5 embryos, we will include them.

3) Fig. 6 would benefit from evidence that MCM3 mutant is rescuing MCM4 levels in the chromatin fraction of cells and the DNA damage phenotype.

The genetic evidence presented is strong, and although we didn’t do the suggested experiment, we feel that our previous studies (McNairn et al., Nature 2019 and Chuang et al., PLoS Genet 2010) on the effects of MCM3 as a nuclear export factor (as it is in yeast (Liku et al., Mol Biol Cell 2005)) are a reasonable basis for not repeating such experiments. Furthermore, we are no longer maintaining the *Mcm3* line and it would take over a year to reconstitute and rebreed triple mutants.
