# Supplementary material for: Chronic replication stress-mediated genomic instability disrupts placenta development in mice
Source: PLoS Genet. 2026 Apr 13;22(4):e1012111. doi: 10.1371/journal.pgen.1012111 (PMC13099090; doi:10.1371/journal.pgen.1012111)
Supplement: Attachment — Submitted filename: Response to ReviewersPGen.docx [file pgen.1012111.s016.docx]

**Response to Reviewers**

We thank the reviewers for providing thoughtful and constructive feedback, which helped us improve the clarity and rigor of the paper. Overall, the reviews were positive. To summarize major opinions: Reviewer 1 mentioned that “This is a strong manuscript with few problems and all important findings well justified, indeed this is a nicely polished…..high-quality manuscript,” and that “this paper makes a major breakthrough, showing that cell autonomous defects in hTSCs are very likely at the heart of the pathology observed in GIN-prone murine mutants.” Reviewer 3 stated that “The study is well designed, and the manuscript is very well written. The conclusions are supported by the evidence presented.” Reviewer 2 differed, stating that “The paper is mostly descriptive and often quite confusing” and felt that figure titles/section headers overstated the results, and recommended improvements to some technical aspects. We carefully considered all the comments, and we feel that the revisions we made – through editing or adding data - have addressed the key issues raised by the reviewers. Our responses to particular points are in RED.

**Reviewer #1**

Summary: 
In a previous paper (McNairn et al. 2019 "Female-biased embryonic death from inflammation induced by genomic instability" Science), the Schimenti lab demonstrated that mouse embryos with hypomorphic mutations of the heterohexameric minichromosome maintenance complex, mutations that cause increased genomic instability (GIN), show reduced embryonic viability, with greater loss of female embryos and some parent-of-origin effect. Treatment with immunosuppressants, including ibuprofen and testosterone, partially rescued the observed lethality. 

In this new manuscript, the Schimenti lab demonstrates that these GIN-prone mutants feature smaller placentas with fewer cells. Mutations that interfere with the ability of the innate immune system to respond to micronuclei (a consequence of GIN) have no protective effect. Munisha and colleagues then demonstrate that MCM-mutant TSCs are harder to derive and show elevated apoptosis and a greater propensity for differentiation. The mutant TSCs show CHK1 phosphorylation, P53 phosphorylation and higher P21 levels, all consistent with a response to DNA damage. Downstream of this, they also show loss and inhibition of CDK1, which is already established to cause G2/M arrest (generally) and endoreduplication (specifically in trophoblast). The authors advance a model in which GIN results in loss of the TSC pool by apoptosis, cell cycle arrest and premature differentiation, resulting in smaller placentas and particularly fewer junctional zone cells. How this causes inflammation is less clear, but inflammation appears to be a downstream effect rather than cause of poor placentation. 

Major comments: 
This is a strong manuscript with few problems and all important findings well justified, indeed this is a nicely polished manuscript for something just entering peer review. There are a few unclear points textually and a couple places in the supplementary figures where better data quality would help, but generally it is a high-quality manuscript.

- I am confused as to the basis of the sex-skewing phenomenon? Is the problem that lack of maternally loaded WT Mcm4 worsens the phenotype, or is the issue that Mcm4C3/C3 dams are less able to retain pregnancies, perhaps being a more inflammatory environment? Also, while there quite consistent evidence for reduced viability of Mcm4C3/C3McmGt/+ progeny, especially for female progeny, how confident can we be that the genotype of the dam vs. sire is important? Notably on a Ddx58 background, the progeny of the Mcm4C3/C3 sire included seven live male Mcm4C3/C3McmGt/+ but no female.

Regarding the first point (sex skewing only when female is Mcm4C3/C3), we also suspected either: 1) the maternal uterine environment, or 2) reduced oocyte quality. Although not reported in this manuscript, we tested #1 by performing embryo transfer experiments. Transferring 2-cell stage embryos from sex-skewing mating to WT females did not rescue the sex-bias. We then examined oocytes from C3/C3 females. We found evidence for compromised mitochondria and transcriptome disruption. However, we are not sure why this happens (poor follicle support? Oocyte intrinsic phenomenon?). We will be reporting these results and additional experiments in a separate manuscript, since this one mainly deals with GIN and placenta development.

Regarding how confident we are that the genotype of the dam vs. sire is important, this stems from our previous paper (McNairn et al 2019), where we found that the percentage of female *MCM4^C3/C3^ Mcm2^Gt/+^* offspring from matings in which the Dam was *Mcm4^C3/C3^* was ~22% compared to 58% when the Dam was *Mcm4^C3/+^ Mcm2^Gt/+^* . A summary of these data have been added to Suppl. Table 1 at the top. Consistent with this, MCM levels were significantly reduced in the placentae only when the dam was *Mcm4^C3/C3^* and the sire *Mcm4^C3/+^ Mcm2^Gt/+^,* but not in the reciprocal cross. The reviewer makes a good observation about the Ddx58 cross; we can only hypothesize that the mutation somehow sensitizes females in this scenario. We also realized that we neglected to write in Methods that the Ddx58 allele was coisogenic in the C3H background; this has been added in the revision.

- I'm not sure what Supplementary Figure 6 is showing (faster differentiation of C3 but less TGC?). Regardless, it's hard to draw too much conclusion from one not-very-pretty Western blot. This figure requires both additional replicates and a better explanation of how it fits with the other conclusions of the paper..

We hypothesized that the JZ defect observed in the semi-lethal genotype placentas could arise either from impaired maintenance of the progenitor pool or from reduced capacity of mutant trophoblast progenitors to differentiate into the JZ lineage. The blot in Supplementary Figure 6 (now Suppl. Fig. 7) was intended as a qualitative demonstration that mutant trophoblast stem cells *can* differentiate into JZ lineages. Unfortunately, the student who generated this data has graduated and the lysates are not available for repeating the Westerns. We believe that defective maintenance of TSCs and premature differentiation leading to depletion of the stem cell pool is the likely explanation for which we have provided several orthogonal forms of evidence - reduced proliferation, increased cell death, reduction of the EOMES-positive stem cell population in semi-lethal genotype TSCs cultured under stem cell culture conditions (Fig. 4), and increased >4C mutant TSCs under stem cell conditions (Fig. 5) - to support this hypothesis.

- Supplementary Figure 7F-G is puzzling. Half of the mESCs have gamma-H2AX at all times, including most in S or G2 phase? In Figure S7E, do the quadrants correspond to being negative or positive for gamma-H2AX? At very least, IF images showing clear gamma-H2AX foci would be much more convincing.

The gates for γH2AX FACS analysis were established using negative controls lacking primary antibody. As reported previously, embryonic stem cells display high basal levels of γH2AX staining (Chuykin et al., Cell Cycle 2008; Turinetto et al., Stem Cells 2012; Ahuja et al., Nat Comm 2016), which likely explains the broad signal observed across cell cycle phases. Nevertheless, as suggested, we replaced the γH2AX flow cytometry data with immunofluorescence staining of γH2Ax and quantified γH2AX foci per nucleus in ESCs. These are now in Suppl. Fig. 8E,F in the revision.

- The methods section is well detailed, but it would be ideal to clarify how many replicates each Western Blot or flow cytometry experiment is representative of.

Thanks for the suggestion. We have updated Fig. 5 with quantifications of the western blots with statistical analysis, and updated the Fig.4 legend reflecting the number of replicates.

*Minor comments:*

- Supplementary Table 1. would be enhanced greatly showing comparable tables for Mcm4C3/C3 x Mcm4C3/+McmGt/+ in mice without the Tmem173 or Ddx58 mutations. It is fine to recycle data from McNairn 2019 here, as long as the source is indicated, but a comparison is needed.

Thanks for pointing this out. We added that data at the top of Supp Table 1.

- Is it possible that cGAS-STING and RIG pathways act redundantly to cause inflammation and lethality, or that other innate immune components are involved? I don't expect the authors to make compound mutants to test this but at least this possibility should be discussed textually.

We appreciate the reviewer’s point, and had the same suspicion. Supporting this, we added RNA-seq analysis which revealed elevated expression of inflammatory genes in *Mcm4^C3/C3^ Mcm2^Gt/+^ Tmem173^-/-^* placentae compared to the controls, consistent with potential redundancy or feedback regulation. This is now reflected in Supplementary Fig.9 and mentioned in the discussion.

- In Figure S3E-F, is the box above each graph supposed to show the genotype of the dam?

Yes. Thanks for pointing this out. We have added a description in the figure legend to make it clear.

- "Indeed, the placenta and embryo weights of E13.5 Mcm4C3/C3 Mcm2Gt/+ Mcm3Gt/+ animals were significantly improved vs. Mcm4C3/C3 Mcm2Gt/+ animals, rendering them similar to Mcm4C3/C3 littermates (Fig. 6A-C). The JZ (but not LZ) area in Mcm4C3/C3 Mcm2Gt/+ Mcm3Gt/+ placentae also increased to the level of Mcm4C3/C3 littermates (Fig. 6D-H)." There are two problems here. First, the figure calls are wrong. Second, the description of the data is not quite right, it looks like the C3/C3 and C3/C3 M2/+ M3/+ LZs are a similar size to each and are statistically indistinguishable.

Thanks for catching this. We have updated the figure callouts and the wording (replaced “(but not LZ)” with “JZ and LZ” in the main text.

Reviewer #2

The manuscript, "Chronic replication stress-mediated genomic instability disrupts placenta development in mice" by Munisha et al follows up a 2019 paper in Nature by the same group where they show that mutations to the MCM genes lead to a sex-skewed semi-lethal phenotype starting after embryonic day 9.5 and extending to birth. In the paper, they hypothesized that the semi-lethality is secondary to genomic instability (GIN) driven inflammation due to activation of the innate immune pathways sensing cytoplasmic DNA. In this paper, they start by disproving that hypothesis and then go on to present data arguing lethality is due to a placental development defect rather than inflammation. The paper is mostly descriptive and often quite confusing leaving one not much closer to understanding the mechanistic basis for the interesting sex-biased semi-lethal phenotype that was described in their original paper. The most interesting aspect of the paper is the derivation of TSC and ESCs and initial analysis suggesting that the TSCs are more sensitive to the MCM mutations, but the analysis is rather shallow. Importantly it is unclear how the phenotype explains the sex-skewing of the phenotype. Are the TSC phenotypes sex-skewed and if so why? Also, why is the JZ and especially GlyTCs most effected?

A major concern throughout the paper is that conclusions are often overstating their data. The title of figure 2 is "placentae with replication stress have smaller junctional and labyrinth zones". However, there is no measure of replication stress in this figure, just a histological evaluation of the placentae from the different mutants. The title of figure 3 is "Impact of GIN on LZ is less than JZ," but there is no measure of GIN, but instead measurement of number of cells in cell cycle and some bulk RNA-seq analysis. Title of figure 4 is "TSCs with increased genomic instability exhibit abnormal phenotypes." Again there is no measure of GIN, but instead staining of derived TSCs for proliferation, cell death, and a TSC marker. Title of figure 5 is "DNA damage responses and G2/M checkpoint activation drive premature TSC differentiation." However, there does not appear to be a difference in gH2AX between the two mutant genotypes. Checkpoint proteins might be up, but need quantification and reproduction. > 4C is the only marker of differentiation. Importantly, all the analyses here are associations, not connections, so cannot use the word "drive". Similar issues can be raised with a number of the supplementary figures.

*Chaos3* (chromosome aberrations occurring spontaneously 3) mice are a well-established model of intrinsic chronic replication stress and GIN. It is characterized by ~20 fold elevation of blood micronuclei (Shima et al., Nature 2007), a hallmark of GIN (Soxena et al., Mol Cell 2022); a destabilized MCM2-7 helicase prone to replication fork collapse (Bai et al., PLoS Genet 2016); and increased mitotic chromosome abnormalities and decreased dormant origins (Kawabata et al., Mol Cell 2011; Chuang et al., Nucleic Acid Res 2012) that are known to cause GIN and replication stress (Ibarra et al., PNAS 2008 ). Nevertheless, we acknowledge that in TSCs, we did not formally demonstrate replication stress (RS), so we revised the titles of figures 2,3,4,5 to refer to “semi-lethal genotype” cells. Similarly, titles of Supplementary Figures have been changed using the “semi-lethal” designation or “replication stress genotype” rather than “replication stress”.

Regarding Fig. 5: In our previous work (McNairn et al Nature 2019), we showed that placentae from C3/C3 dams exhibit significantly elevated γH2Ax as well as reduced MCM2 and MCM4 protein levels. In our current study, we also observe elevated γH2Ax in mutant TSCs (C3/C3 and C3/C3 M2/+) compared to WT (Fig. 5A,B), consistent with genomic instability. We acknowledge the reviewer’s concern regarding western blots. We have provided quantification and statistics in our revision for Fig5.

Major Comments:

1)  A deeper analysis of the cell lines is likely to be the most fruitful path to reveal interesting mechanisms. It is very surprising that there is no phenotype in ESCs. Authors should check for increased apoptosis. Maybe the phenotypic cells are lost. Or do ESCs use different MCMs/mechanisms of DNA replication or are they better able to handle replication stress and GIN? How many passages were the TSCs and ESCs cultured for? Does GIN (i.e. aneuploidy, CNVs) develop in TSCs and ESCs with passaging? How do the MCM mutations impact the molecular identity of the ESC and TSC cells including their heterogeneity in the population.

We assessed apoptosis using cleaved caspase 3 flow cytometry in mutant ESCs and observed no difference compared to controls (now presented in Supplementary Fig. 8G).

We believe there are intrinsic differences in TSCs and ESCs in their ability to respond to and counteract replication stress and DNA damage. ESCs are known to license more replication origins than somatic cells at a higher rate, which protects them from short G1-induced replication stress (Ahuja et al., Nat Comm 2016; Ge et al., Stem Cell Rep 2015; Matson et al., eLife 2017). Human placental cells physiologically exhibit high levels of mutation rate and chromosomal instability *in vivo* (Coorens et al., Nature 2021)*.* Supporting this, Wang, D., et al (Nat Comm 2025) reported that several cell cycle and DDR regulators are differentially expressed in human TSCs vs human pluripotent stem cells. Whether such transcriptional differences directly contribute to functional outcomes remains to be determined.

All experiments in this study were conducted using early-passage ESCs and TSCs (i.e. <10 passage) that we ourselves derived. Although we agree that examining mutation rates with extended passaging is of interest, this is beyond the scope of the current work and would necessitate major new experimentation such as WGS of subclonal lines over time.

Finally, we showed that close to 90% mutant ESCs are KLF4+ (a naive pluripotency marker) whereas EOMES+ cells were significantly reduced in TSCs carrying the GIN genotype (Fig. 4E–F and Supplementary Fig. 8), highlighting lineage-specific differences.

2) Along similar lines, most of the in vivo phenotypic analyses are performed at E13.5, long after defects are likely beginning to express themselves especially given that they see phenotypes in the TSCs, which represent the polar TE of a E4.5. To understand the primary defects of the in vivo phenotype, they should be looking much earlier. Supplemental figure 5 is a start but represents a rather superficial analysis.

The peri-implantation period, namely E4.5, represents a “black box” of embryonic development and is extremely difficult stage to analyze technically. We don’t think it is essential to the conclusions (or doable in a timely manner, given that compound mutants for both sexes would be needed and the colony would have to be expanded to enable sacrifice of females), especially given the use of TSCs.

3) Fig. 6 would benefit from evidence that MCM3 mutant is rescuing MCM4 levels in the chromatin fraction of cells and the DNA damage phenotype.

Although we didn’t do the suggested experiment, we feel that our previous studies (McNairn et al., Nature 2019 and Chuang et al., PLoS Genet 2010) on the effects of MCM3 as a nuclear export factor as it is in yeast (Liku et al., Mol Biol Cell 2005) are a reasonable basis for not repeating such experiments. Furthermore, we are no longer maintaining the *Mcm3* line and it would take over a year to reconstitute and rebreed triple mutants.

Minor Comments:

1) There is a lack of quantification and repeats for all Westerns. At minimum there should be three repeats for each experiment, quantification including normalization to a reference protein, and stats confirming any proposed differences between conditions.

We have updated our revision with quantification and statistics for western blots for Fig4. Quantifications were performed using biological replicates, i.e. different cell lines from different genotypes.

2) I would recommend moving the results in supp table 1 to figure 1. While negative, they are the newer results. The results shown in current figure 1 are essentially a reproduction of their previous work.

The placental observations presented in Fig.1 are new. In particular, the placental and embryonic weight measurements graphed in Fig1B and C have not been published by our group. Fig1A schematically summarizes our previous observation on embryo viability in GIN mutants (McNairn et al., Nature 2019), which we included to hopefully orient readers given the complex mating schemes. We are agnostic on the Suppl Table 1. It could be changed to a new Table 1 in the main section if desired.

3) Need to review citations to figures. For example, no citations are made to figure 4a and 4c.

Thanks for catching this. We have updated the text.

**Reviewer #3**

This manuscript examines chronic replication stress-mediated genomic instability in placental development and concludes that it disrupts placental development in mice. The study is well designed and the manuscript is very well written. The conclusions are supported by the evidence presented. The manuscript would be improved by addressing the comments below.

Major Comments

• While the inclusion of bulk RNAseq data of whole placental tissue is appreciated, the interpretation of the results is somewhat problematic, as it is acknowledged that the cell type composition of the placentas is drastically different between groups. Making conclusions based upon GSEA analysis of two different groups with drastically different cell type composition is somewhat misleading, as based on the results, it is a direct reflection of the cell types present. It would be more helpful to perform cell type deconvolution of the RNAseq data to estimate the proportion of each cell type within the bulk samples and compare that to what is seen histologically and not dive too deeply into the pathways since the results could just be a reflection of the cell types e.g. angiogenesis pathways from more endothelial cells. Additionally, the RNAseq data can be leveraged to look at expression of inflammatory genes by sex, which may show interesting patterns based on the other results.

We agree that the cellularity differences between control and mutants placentae is complicating especially for underrepresented genes. We performed deconvolution of the bulk RNA-Seq using publicly available E13.5 scRNA-Seq data using the BayesPrism tool (Chu et al., Nat Cancer 2022). We did observe an overrepresentation of endothelial cell types in deconvoluted mutant bulk samples, indicating that the overrepresentation of the vasculogenic pathway was indeed a reflection of the abundance of endothelial cells. We have added these observations in Supplementary Fig4, and updated the main text accordingly.

• Section: GIN impairs trophoblast stem cell establishment and maintenance. To support the assertion in the first paragraph, beyond measuring apoptosis, it would be helpful at this stage to look at RNA expression levels indicative of the activation of DNA damage checkpoint genes

Thanks for the comment. We realize now that the first sentence of this section, now entitled, “Semi-lethal genotypes impair trophoblast stem cell (TSC) establishment and maintenance”, was misleading because it invoked the DDR (“In most cell types, high levels of DNA damage result in the activation of DNA damage checkpoints causing cell cycle arrest or apoptosis”). However, the issue of checkpoint is actually addressed in the following section of Results, where we present evidence of checkpoint activation (phosphorylation of key checkpoint proteins) as shown in Fig. 5. We deleted the aforementioned sentence.

• Define the first use of >4C DNA content to help readers understand this potentially unfamiliar term.

We have edited this part to indicate cells with more than 4C DNA content for better clarity.

• Please include additional methodological details in the methods section on the statistical analysis done for differential expression analysis. Specifically, what type of normalization was used, if lowly expressed genes were filtered out and at what cutoff, what statistical model was used (did you include covariates?), what comparisons were made? Did you stratify by sex? What cutoff was used for statistical significance? Did you perform multiple testing correction?

Thanks for pointing out. We have elaborated in the Materials and Methods subsection: “Bulk RNA-seq and data analysis”. See the new highlighted partsin the markup version of the ms.

• iDEP tool - please include citation to manuscript instead of link

Done.

• Check citations. Some citations to BioRxiv that are now published e.g. 13.

Done.
