# Supplementary material for: Chronic replication stress-mediated genomic instability disrupts placenta development in mice
Source: PLoS Genet. 2026 Apr 13;22(4):e1012111. doi: 10.1371/journal.pgen.1012111 (PMC13099090; doi:10.1371/journal.pgen.1012111)
Supplement: Attachment — Submitted filename: Response to Reviewersv3.docx [file pgen.1012111.s017.docx]

We would like to thank the reviewers for their timely and insightful reviews of the initial and revised versions of our manuscript. They were instrumental in helping us hone the quality of the report.

Response to Reviewer #1: The reviewer was satisfied with the previous revision.

Response to Reviewer #2: (in red)

• On Page 10: “Analysis of publicly available single-cell RNA-Seq (scRNA-Seq) data from E13.5 mouse placenta revealed trophoblast and non-trophoblast cell types based on the most differentially expressed as well as previously known marker genes (S4A-B Fig).” This sentence does not contain information beyond what single cell analysis is, so it is not helpful to include. It is also unclear why S4A-B were included in this manuscript, as A and B are previously reported in the data source publication. If these are distinct, please include the details of the process of the annotations and how they are different from the source.

The reviewer’s comment made it evident that the indicated sentence was unclear and not very informative, so the beginning of the paragraph was condensed to emphasize the rationale for gene expression experiments (to confirm histological data) and the analyses performed.

Regarding the inclusion of S4A-B and our re-analysis the published data, there were two important reasons: 1) The original Jiang et al., Cell Discovery (2023) paper did not include a separate analysis of trophoblast cells in the E13.5 placenta; they integrated multiple developmental time points for downstream analysis. Therefore, we found it necessary to match the developmental time point of the source scRNA-Seq data from Jiang et al with our bulk RNA-seq data. 2) The single cell reference for BayesPrism requires character vectors that denote the cell types (cell.state.labels) in the reference Seurat objects. Hence, we needed to re-analyze the published pre-processed E13.5 raw count matrix. Parameters for annotating the cell types were added in the Methods subsection entitled “Bulk RNA-seq deconvolution”.

• Supplementary Fig 4C that includes the pairwise correlation of cell types by gene expression is unnecessary and does not add additional information relevant to this manuscript.

We appreciate and understand this comment. However, whereas some trophoblast cells such as p-TGC and S-TGC are distinctly clustered in the UMAP, their highly similar transcriptional profiles prevented BayesPrism from differentiating the proportions of these cell types. Thus, most transcripts were fed into one of the cell fractions (for example, the extremely small fraction of S-TGCs compared to the p-TGCs). We included Supp Fig 4C as an explanation for this limitation of BayesPrism analysis. Thus, we think it is important to leave in this panel.

• Cell type deconvolution statistics between groups is done with incorrect statistical tests for comparing proportions. Please revise statistical test and restate conclusions on page 10. Also include pvalues for significant tests in the manuscript.

We updated the statistical test using a generalized linear hypothesis. We reported the Benjamini–Hochberg FDR adjusted q-value in our revision.

• Concerns remain for the bulk RNAseq analysis and statement of results. Given the difference in endothelial cell types between conditions, the bulk rnaseq analysis results are skewed by cell type. Please re-run the bulk RNAseq differential analysis including cell type proportions for those that are significant as covariates to remove the effect of those imbalances on the results. While running with covariates, it would also be helpful to run a model that includes both sexes and include sex and cell type proportion as covariates (e.g. ~0 + condition + endothelial_cell_proportion + sex). Limma-voom may be a more flexible tool for this model than DEseq2. Note: the inclusion of many covariates may be limited by sample size, so

We understand the reviewer’s concern. The purpose of GSEA in Fig3 and FigS5 was to provide orthogonal support for the underlying anatomical defects observed in C3/C3 M2/+ placentae. The deconvolution was in agreement with GSEA analysis.

However, given the reviewer’s concern, we performed differential analysis using the suggested model where the endothelial cell proportion was treated as a co-variate. When we analyzed gene sets involved in placenta development using the following model: [~0 + Genotype + Endo + Sex] vs [~0 + Genotype + All + Sex] in which “All” represents all cell type proportions that are significant, the labyrinth related pathways changed more drastically, and sex differences were abolished (see below and Fig3C). Under-representation of major placenta-related pathways remained under-represented in females confirming our structural observations. Therefore, we updated Figures 3C and D (as well as FigS8) and the main text with the results obtained from model [~0 + Genotype + All + Sex] to reflect the above-mentioned changes. We also observed that sex differences involved in angiogenesis pathways were abolished. Therefore, we deleted Fig S5 and related conclusions in the main text.

• Report pvalues in the text for all of the statements that report a significant difference e.g. Page 15 “western blot analysis revealed elevated levels...”

Thanks for pointing this out. We have added the P-values directly to the figures, replacing asterisks denoting ranges. These changes are in all 6 main figures, plus supplementary Figs 1,2,3 and 5.

Specific Comments:

• Supp Figure 4D: include jitter in plots for individual samples. What does “mean fraction of cell types” represent? Shouldn’t there be a single fraction for each sample, so you are not reporting the mean?

Thanks for catching this. Y-axis is the average/mean cell type fraction from each placental sample. We have added dots to the plot to indicate replicates.

• Page 27: sentence fragment “SARTools”

We deleted this partial sentence as it was a leftover from previous edit. Thanks for catching that.

• Add software version numbers for Cutadapt, FASTQC, Fastx, iDEP, DESeq2

Versions were updated in the Methods section.

• Page 28 top – include a few more words for clarity of quantifying the bulk samples.

Added a highlighted section on page 28 for clarity of deconvolution.
